# The Role of Calprotectin in Pediatric Disease

**DOI:** 10.1155/2013/542363

**Published:** 2013-09-23

**Authors:** George Vaos, Ioannis D. Kostakis, Nick Zavras, Athanasios Chatzemichael

**Affiliations:** ^1^Department of Pediatric Surgery, Alexandroupolis University Hospital, Democritus University of Thrace School of Medicine, Dragana, 68100 Alexandroupolis, Greece; ^2^Second Department of Propedeutic Surgery, “Laiko” General Hospital, National and Kapodistrian University of Athens, Medical School, 17 Agiou Thoma Street, 11527 Athens, Greece; ^3^Third Department of General Surgery, “Attiko” General Hospital, National and Kapodistrian University of Athens, Medical School, 1 Rimini Street, 1246 Athens, Greece; ^4^Department of Pediatrics, Alexandroupolis University Hospital, Democritus University of Thrace School of Medicine, Dragana, 68100 Alexandroupolis, Greece

## Abstract

Calprotectin (CP) is a calcium- and zinc-binding protein of the S100 family expressed mainly by neutrophils with important extracellular activity. The aim of the current review is to summarize the latest findings concerning the role of CP in a diverse range of inflammatory and noninflammatory conditions among children. Increasing evidence suggests the implication of CP in the diagnosis, followup, assessment of relapses, and response to treatment in pediatric pathological conditions, such as inflammatory bowel disease, necrotizing enterocolitis, celiac disease, intestinal cystic fibrosis, acute appendicitis, juvenile idiopathic arthritis, Kawasaki disease, polymyositis-dermatomyositis, glomerulonephritis, IgA nephropathy, malaria, HIV infection, hyperzincemia and hypercalprotectinemia, and cancer. Further studies are required to provide insights into the actual role of CP in these pathological processes in pediatrics.

## 1. Introduction 

Calprotectin (CP) is a calcium- and zinc-binding protein of the S100/calgranulin family [1]. It is also referred to in the literature as S100A8/A9, MRP8/14 (myeloid-related protein), calgranulin A/B, L1 protein, 27E10 antigen, cystic fibrosis antigen, myeloid-histiocyte antigen, and CP-10 (the last refers to the light chain only) [1–3]. CP is mainly exhibited in the cytoplasm of neutrophils (about 5% of their total protein contents [1, 4] and 30–60% of their cytosolic protein [2]). It is not only expressed on activated monocytes and macrophages (about 1% of all monocyte cytosol protein) [3, 4] but can also be produced by bone marrow cells, squamous epithelium (keratinizing and nonkeratinizing), some mucosal epithelial cells, microvascular endothelial cells, fibroblasts, generally as a result of activation [1, 2, 5]. CP has antibacterial [1–3, 6], apoptosis-inducing [1–3, 6, 7] and chemotactic activities [1, 7–9]. It also participates in leukocyte interactions with the endothelium [2, 3, 10], cellular adhesions leading to the recruitment of leukocytes to inflamed intestinal tissue [1–3, 10, 11], and with the inflammatory [1, 3, 6–8, 10] and thrombogenic response of endothelial cells [3, 10]. Elevated plasma levels of CP are evident in infectious and inflammatory diseases [4]. 

 CP is a 36.5-kDa heterodimer composed of one light (MRP8) and two heavy (MRP14) calcium-binding chains (8-kDa, S100A8/L1L/p8/CP-10 and 14-kDa, S100A9/L1H/p14). CP also contains zinc-binding sequences (His-X-X-X-His motif) involved in its antibacterial activity [2]. Additionally, it can be identified as a monomer, with separate chaining, or as a hetero- or homodimeric, trimeric or tetrameric complex [12]. The genes for calprotectin are located on the human chromosome 1q21 [1]. 

 There are several advantages to the use of CP as an inflammatory marker in pediatric diseases [2]. Fecal calprotectin (FCP) is an objective and non-invasive test reflecting various pathological processes occurring in the mucosa of pediatric patients. Sieric calprotectin (SCP) could be a sensitive nonspecific inflammatory marker in various pediatric conditions. Another relevant clinical issue concerns the determination of calprotectin-positive monocytes/macrophages in a tissue. Albeit this method requires a biopsy, it can be useful for evaluating the invasion of mononuclear phagocytes at the site of inflammation. This paper aims to review the role of calprotectin in a range of inflammatory and other pathological conditions among pediatric patients. 

## 2. Fecal Calprotectin 

### 2.1. Inflammatory Bowel Disease

FCP concentrations represent bowel inflammation in children with IBD [13]; elevated values are observed in both Crohn's disease and ulcerative colitis cases [14]. However, optimal cut-off values have yet to be determined in pediatric patients although a cut-off level of 50 *μ*g/g appears to be the most proper cut-off point for the FCP test [15]. A positive result supports the diagnosis or relapse of IBD, but a negative result does not necessarily exclude it [16]. The effectiveness of FCP seems to be moderate in predicting subclinical relapse in IBD. Further research on evidence-based medicine is required to understand the significance of FCP in predicting IBD relapse. An FCP concentration test may also prove useful in children since it is higher in IBD as compared to children with other types of IBD, such as lymphocytic, eosinophilic, and nonspecific colitis [17]. Preliminary evidence shows that CP is also present in elevated concentrations in the colonic mucosa of children with IBD and may participate in its pathogenesis [18]. In the presence of gastrointestinal symptoms, FCP can be helpful as a noninvasive tool in the prediction of pediatric colorectal inflammation in combination with other clinical and laboratory indices, such as a fecal occult blood test, which is less likely to be more positive than CP, C-reactive protein (CRP), fecal lactoferrin, and clinical disease activity [14, 19–22]. It facilitates recognition of apparent clinical and laboratory remission and is useful for assessing more accurately the severity of mucosal inflammation as compared to other clinical and laboratory indices [20, 23–27]. FCP normal levels represent complete mucosal healing [28]. FCP is also useful for identifying children who are most likely to need endoscopy requiring general anesthesia for suspected IBD, thereby reducing the need for referrals, and it has a low risk of missing cases. However, the specificity is significantly better in studies of adults than in studies of children [29–33]. Finally, children with functional bowel disorders and noninflammatory diseases display FCP values within normal range [34]. Other studies have reported increased levels of FCP in children with functional abdominal pain and irritable bowel syndrome that correlated with the extent to which pain interfered with activities rather than stool form [35]. 

 While treatment of active IBD with glucocorticoids leads to a decrease in FCP concentrations parallel to clinical improvement, levels fail to return to normal; this indicates continuing inflammatory activity in a clinically silent disease [36]. Administration of primary nutritional therapy with exclusive enteral formula feeds (polymeric semi-elemental, or elemental formula) for eight weeks induces clinical remission and reduces inflammatory activity in the intestinal mucosa [37]. Dietary therapy has also been used in other inflammatory bowel conditions except IBD. A recent study showed that the addition of *Lactobacillus rhamnosus* GG to an extensively hydrolyzed casein formula improved the recovery of the inflamed colonic mucosa in infants with allergic colitis, as indicated by the significant decrease in FCP and improvement of hematochezia after one month [38]. Moreover, treatment with infliximab-a TNF-*α* antagonist- has been shown to decrease FCP concentrations and reach normal levels at 2 weeks in one third of pediatric patients with IBD, reflecting mucosal healing [39].

 Although FCP is an inexpensive, easy, specific, and sensitive test in the assessment of IBD, and while it plays an important role in the diagnosis, followup, assessment of relapses and response to treatment, it is not without its disadvantages and it seems that it can only be used as a complementary test.

### 2.2. Diarrhea

FCP may be used as a useful fecal inflammatory marker in helping to distinguish between constitutive and immune-inflammatory causes of severe persistent diarrhea of small children [40]. Infectious diarrhea causes significantly higher FCP concentrations than those displayed in irritable bowel syndrome (IBS) which are comparable with the values found in healthy controls. FCP levels correlate with the clinical severity of infectious diarrhea in children [41]. Children with Crohn's disease also have higher FCP values than children with IBS or infectious diarrhea. Furthermore, FCP displays high sensitivity in cases of food intolerance while, in identifying organic causes of chronic diarrhea, CP shows better sensitivity and specificity in children than in adults [42]. 

### 2.3. Juvenile Idiopathic Arthritis

A recent study showed that FCP may be used to evaluate the subclinical intestinal inflammation in children with juvenile idiopathic arthritis (JIA) though the study in question had several limitations [43]. Further studies are warranted to confirm the actual role of elevated FCP in those children. 

### 2.4. Necrotizing Enterocolitis

FCP may be a useful marker for necrotizing enterocolitis (NEC) since raised FCP concentrations exceeding 350 *μ*g/g are detected and followed by bowel perforation, bloody stool, and other clinical features of NEC representing signs of gastrointestinal injury [44, 45]. Moreover, it has been found that FCP decreases as NEC resolves. However, the usefulness of FCP as such a marker may be controversial since high interindividual variations in healthy full-term and preterm infants and high CP concentrations in healthy neonates during the first month of life have been observed [44]. A correlation between FCP and severity of NEC in preterm infants has also been reported [46]. Furthermore, the combination of FCP and intestinal fatty acid-binding protein seems to improve the diagnostic accuracy in infants with suspected NEC early on in the disease [47]. On the contrary, some authors advocate that FCP does not play a role in the diagnosis of NEC, particularly in the early stages of disease [48]. Larger prospective studies of patients are needed to demonstrate whether FCP has a role to play in NEC as a potential biomarker. 

### 2.5. Celiac Disease

Data on FCP in celiac disease (CD) are still limited. FCP concentration may be useful in the diagnosis and followup of children with CD. Increased FCP concentration in untreated CD patients returns to normal on a gluten-free diet [49]. A relationship between FCP concentration and the severity of histopathologic findings has also been documented in childhood CD [50]. The mechanism by which FCP levels increase should be further researched. 

### 2.6. Intestinal Cystic Fibrosis

The effects of intestinal inflammation on the nutritional and pulmonary status in cystic fibrosis (CF) have not been fully researched until now. Future investigation is required to define the most reliable biomarkers of intestinal inflammation in CF. A controlled, prospective study showed that children with CF display significantly elevated FCP concentrations, which fall after probiotic administration [51]. However, further studies are required before probiotic administration can be routinely used in children with CF. Very recent findings also suggest that FCP has a promising role to play in the diagnosis of these patients [52]. 

### 2.7. HIV Infection

Human immunodeficiency virus-infected, highly active antiretroviral therapy-naïve Ugandan children above 4 years of age have a median FCP concentration above the reference value, while patients with advanced disease have raised FCP concentrations regardless of age [53]. 

## 3. Sieric Calprotectin 

### 3.1. Inflammatory Bowel Disease

Apart from FCP, sieric calprotectin (SCP) is also elevated in children with IBD, possibly indicating clinical disease activity in these patients [18]. 

### 3.2. Necrotizing Enterocolitis

A very recent prospective multicenter study showed that SCP may be an accurate marker for early diagnosis of NEC in neonates. Defining the cut-off value at 30 mg/mL the accuracy of CP for diagnosis of NEC was estimated: sensitivity 100%, specificity 96.4%, positive predictive value 88.9%, negative predictive value 100%, and likelihood ratio for positive test 28 [54]. 

### 3.3. Juvenile Idiopathic Arthritis

CP has evolved as an excellent biomarker of inflammatory processes in JIA. Concentrations of SCP are significantly increased in children with an active systemic-onset JIA, supporting its use as a diagnostic tool for systemic-onset JIA and allowing these patients to be distinguished from those with other inflammatory diseases [55]. SCP levels are also significantly higher than those recorded in children who have been in stable remission for one year [56]. Furthermore, SCP values may identify children with an increased risk of relapse and unstable remission [57]. In children with oligoarticular and polyarticular JIA, elevated SCP levels indicate residual activity, even in the absence of laboratory or clinical signs of continuing inflammation. Moreover, normal SCP levels in clinical inactive JIA could be useful in identifying those patients in remission in whom methotrexate treatment can be withdrawn [58]. There is also a general activation of the cutaneous epithelium confirmed by the expression of MRP8 and MRP14 genes. Leukocytes also infiltrate the epithelium of sweat gland ducts and MRP8, but not MRP14, expressed by the secretory cells of sweat glands during active JIA [59]. Finally, SCP levels in JIA and the Child Health Assessment Questionnaire (CHAQ) and erythrocyte sedimentation rate are positively correlated though this does not apply to the total leukocyte count, after an autologous hematopoietic stem cell transplantation (ASCT) for refractory JIA. Within the first three months of ASCT, mean SCP concentrations greatly decrease and CHAQ and other clinical parameters of disease activity markedly improve, while SCP concentrations increase during transient relapses [60]. 

### 3.4. Kawasaki Disease

Levels of SCP and those of mRNA of MRP8 and MRP14 in granulocytes are markedly increased in the early stages of acute Kawasaki disease in infants and young children, and they rapidly decrease within 24 hours of intravenous administration of immune globulin. This refers to responders, while both sieric and mRNA levels continue to rise after the initial treatment [61, 62]. Increased percentages of calprotectin+/tumor necrosis factor-alpha+ monocytes in patients with acute Kawasaki disease have also been reported to be the result of monocyte activation by certain peptides derived from oral streptococci [63].

 Later development of coronary aneurysms is observed in children with persistent elevation of SCP after intravenous administration of immune globulin [62]. Furthermore, within two weeks of the onset of symptoms, patients with acute Kawasaki disease have more CP-positive circulating endothelial cells in their blood than control patients, especially those who develop coronary artery lesions. Thus, the concentrations of SCP may be used as markers of disease activity, and the number of CP-positive circulating endothelial cells may reflect the severity of vasculitis, as a result of distinct inflammatory reactions in the endothelium [61]. 

### 3.5. Respiratory System

In children, genes related (S100A8 and GAS6) and unrelated (CD200 and RBP7) to infections are differentially expressed during asthma exacerbation as confirmed by using quantitative real-time RT-PCR [64]. Potential regulation of the expression of MRP8 and MRP14 mRNAs in CF transmembrane conductance regulator protein in CF human tracheal gland cells has been reported [65]. Overall, SCP levels in children with CF, many of whom have infective pulmonary exacerbations, are significantly higher than those seen in healthy controls and reflect the extent of inflammation in these children [66]. 

### 3.6. Acute Appendicitis

There is a loss of cytoplasmic immunoreactivity for S100A8 in vivo in acute appendicitis, which characterizes phagocytic activation of neutrophils and may be useful as a marker of localized neutrophil activation in tissues [67]. CP could be a potential and new diagnostic test for acute appendicitis in adults and children [68, 69]. However, there is no clinically relevant correlation between SCP and the classical tests for  CRP and white blood cell count (WBC). Very recently, it was reported that SCP is increased in children with acute appendicitis and in children with a perforated appendix as compared to those with no perforated appendix. However, the WBC count performed better than CP in the diagnosis of acute appendicitis [70] although poor specificity was observed with WBC and CP. There is currently no evidence to support that SCP is superior to classical inflammatory markers to confirm or exclude suspected appendicitis in children with abdominal pain [71]. 

### 3.7. Hyperzincemia and Hypercalprotectinemia

A new disease which includes recurrent infections, hepatosplenomegaly, anemia, evidence of systemic inflammation and increased CP levels is characterized by dysregulation of zinc metabolism combined with raised CP concentrations in plasma [72]. A possible heritable disorder of CP metabolism was observed in an infant with hypercalprotectinemia/hyperzincemia and systemic inflammation [73]. There is also a reported case of microcytic anemia and inflammation caused by an inborn error of zinc metabolism due to a dysregulation of CP metabolism [74]. Cyclosporine A has been shown to be effective against hyperzincemia and hypercalprotectinemia [75]. Treatment with Tacrolimus seems to have only a transient effect; despite an initial improvement in clinical and biological markers, hyperzincemia, and hypercalprotectinemia progressively worsen [76]. Another report recorded a significant improvement in a patient with hyperzincemia and hypercalprotectinemia following the use of an IL-1 inhibitor [77]. 

### 3.8. Malaria

Mean concentration of SCP of children with high parasitemia is more than four times higher than that of children with low parasitemia. Fever is also detected more frequently in the former group than in the latter. SCP values in children exposed to *Plasmodium* are higher than those of children who have not been exposed. Moreover, it has been observed that SCP values are closely related to parasitemia and fever episodes. Thus, even if increased SCP levels in the blood are not specific to malaria, they could be useful in estimating malaria-related morbidity [78]. 

## 4. Calprotectin-Positive Monocytes/Macrophages

### 4.1. Glomerulonephritis

Infiltrating macrophages in the glomeruli produce MRP8 and MRP14 proteins and form MRP8/14 complexes in correlation with the severity of inflammatory response and activity of glomerulonephritis, as shown by immunohistochemical analysis of renal biopsies from different forms of glomerulonephritis, some of which include a relatively high portion of juvenile patients [79]. In contrast, a great number of macrophages in the renal interstitium produced MRP8 and MRP14 without forming their complex, indicating a chronic inflammatory response in glomerulonephritis. It has been revealed that different macrophage subpopulations are prevalent in different types of glomerulonephritides due to the variety of inflammatory mechanisms involved in glomerulonephritides [79].

 It has also been demonstrated that positive MRP8 staining in glomeruli and the interstitium is significantly higher in cases of persistent nephropathy and renal insufficiency than in cases of minor urinary abnormalities. Moreover, MRP8 production in macrophages in glomeruli and the interstitium in the first biopsy can be used as a prognostic marker for renal dysfunction in children with membranoproliferative glomerulonephritis type 1 [80]. 

### 4.2. IgA Nephropathy

In children with IgA nephropathy and normal urine or with minor urinary abnormality, the accumulation of macrophages expressing MRP8 in glomeruli is higher in specimens from the first biopsy than those from the second biopsy. On the other hand, in children with persistent IgA nephropathy, the accumulation of macrophages expressing MRP8 in glomeruli found in specimens from the first and second biopsy is comparable. The indices of renal sclerosis, which are higher in second biopsy specimens in cases of persistent IgA nephropathy as compared to the first, are higher when there are more macrophages producing MRP8 than when there are less. Thus, renal macrophages producing MRP8 may play an important role in the development of sclerotic changes in cases of children with IgA nephropathy [81]. 

### 4.3. Polymyositis

In polymyositis, MRP14 is expressed by the majority of macrophages detected primarily in the endomysium as shown in a study including predominantly children [82]. 

## 5. Oncology

CP may play a role as an innate amplifier of inflammation in cancer development and tumor spreading [83, 84]. In children, high levels of CP are expressed by bone marrow-infiltrating metastatic neuroblastoma cells. Hence, CP may represent a novel diagnostic marker and potential target for therapeutic intervention in high-risk neuroblastoma patients [85]. More research is needed to define whether the expression of CP by bone marrow-infiltrating neuroblastoma cells is acquired or transcriptionally regulated. 

## 6. Future Perspectives

At present, growing clinical experience shows an expanded role for FCP in diagnosis, the monitoring of remission and mucosal healing, and in the prediction of relapse in pediatric IBD. Nevertheless, there are still questions concerning the reliability of FCP, especially in the field of mucosal healing. However, FCP will identify children at risk of an IBD and will also decrease diagnosis delays and the need for invasive tests such as colonoscopy. Recent studies showed that the diagnostic accuracy of SCP is greater than that of conventional inflammatory markers in IBD or other inflammatory diseases. Larger prospective analyses are required to confirm these findings and to assess better therapy strategies and long-term outcome based on noninvasive measurements of CP. Future studies might show whether changes in CP levels can be of prognostic significance for hospital stay, the need for surgery, and impact on the quality of life. The challenge now is to perform genetic association studies which may show the relationship between genetic variation in A100A8/S100A9 and risk of diseases and might be used as a prognostic marker in the future. 

 Although SCP is increased in children with active JIA, more studies are needed to determine whether SCP levels can predict further damage in those patients. Moreover, specific blocking of pro-inflammatory mediators such as CP achieves improvement and remission in children with JIA. Future controlled studies have to define risk factors of therapy-resistant courses of the disease and establish long-term stable remission in JIA at the early stage of the disease. An understanding of the biomarkers and pathological mechanisms during this early stage would possibly determine new therapeutic strategies and ensure optimal therapy for individual patients. 

 The identification of pretumor clones may provide useful biomarkers of tumor development risk. Patients having these clones may potentially be at greater risk of tumor development and treated differently to those with a less prolific clone. 

 CP, a reproducible and clinically important marker, may have a more meaningful place in future diagnostic and therapeutic pathways. Further detailed molecular research may result in the discovery of new biomarkers that could prove valuable in pediatric clinical practice. Such biomarkers may have a significant role to play in the management of pediatric disease in terms of disease risk assessment, outcome prediction, early appropriate therapy, and possibly the prevention of disease. 

## 7. Conclusion

CP is an important pro-inflammatory mediator in acute and chronic inflammation. Over the last few years, the pivotal role of CP in inflammatory pediatric diseases has been progressively appreciated. The current literature shows that CP may be useful as a marker of inflammatory disease activity and could, therefore, be implicated in the diagnosis and treatment of a variety of inflammatory and other pathological conditions in pediatric patients. More specifically, the FCP test provides higher sensitivity, specificity, and predictive value and performs better than other tests in the evaluation of pediatric IBD. FCP values play an important role in disease assessment and monitoring of children with IBD and could predict disease clinical course. However, they could only be used as a complementary test till now. More recently, increased CP levels were expressed in neoplastic pediatric tumor cells. Despite many possible functions of CP, its biological role still remains unclear. Further studies are needed to elucidate the clinical relevance of CP in various pathological pediatric conditions and to establish whether CP has any implication beyond that of an inflammatory mediator. 
